# Transposase-Associated Variation near *tnaA* in *Porphyromonas gingivalis* Is Linked to Indole Production and Virulence-Associated Gene Expression

**DOI:** 10.3390/pathogens15060617

**Published:** 2026-06-09

**Authors:** Li Wei, Chengjia Xie, Qingnan Ren, Mengfan Zhi, Song Shen, Xiufeng Gu, Qiang Feng, Tianyong Sun

**Affiliations:** 1 Department of Human Microbiome, School and Hospital of Stomatology, Cheeloo College of Medicine, Shandong University & Shandong Key Laboratory of Oral Diseases & Shandong Engineering Research Center of Dental Materials and Oral Tissue Regeneration & Shandong Provincial Clinical Research Center for Oral Diseases, Jinan 250012, China; weilimail@mail.sdu.edu.cn (L.W.); xiecj_cherry@126.com (C.X.); dreamzmf@outlook.com (M.Z.); shensong@sdu.edu.cn (S.S.); 202062070617@sdu.edu.cn (X.G.); 2 Department of Vascular Surgery, General Surgery, Qilu Hospital of Shandong University, Jinan 250012, China; 202315578@mail.sdu.edu.cn; 3 Shandong University-BOP Joint Oral Microbiome Laboratory, Jinan 250012, China

**Keywords:** comparative genomics, periodontitis, *Porphyromonas gingivalis*, indole, tryptophan metabolism, virulence

## Abstract

Indole, a volatile metabolite produced by bacterial tryptophanase (encoded by *tnaA*) during tryptophan metabolism, contributes to oral malodor and may influence the progression of periodontitis. However, the genetic features underlying strain-specific indole production and its association with bacterial virulence remain unclear. Analysis of a previously published periodontitis cohort revealed that periodontitis severity was associated with salivary indole-related metabolic signatures, which were positively correlated with the abundance of *Porphyromonas gingivalis* (*P. gingivalis*). Further analysis showed that W83, a reference strain previously reported to exhibit relatively high virulence-associated characteristics, produced significantly higher levels of indole than ATCC 33277 under the experimental conditions. Comparative genomic analysis of 36 complete *P. gingivalis* genomes showed that the amino acid sequences of TnaA were highly conserved. However, the transposase region adjacent to *tnaA* differed among strains: previously reported high-virulence strains, including W83, W50, and A7436, harbored the IS5-family transposase ISPg1, whereas several low-virulence reference strains carried the IS982-family transposase IS195. In saliva samples from periodontitis patients, ISPg1 expression was positively correlated with *tnaA* expression, and both were associated with periodontal clinical parameters. Together, these findings indicate that transposase-associated genomic variation near *tnaA* is associated with strain-specific tryptophan-indole metabolism, virulence-associated gene expression, and periodontal clinical parameters, while direct causality remains to be established in future functional studies.

## 1. Introduction

Periodontitis is a chronic inflammatory disease characterized by the destruction of tooth-supporting tissues, frequently accompanied by oral malodor or halitosis [1]. Halitosis mainly arises from malodorous metabolites produced by oral bacteria, including indole [2,3,4], volatile sulfur compounds (VSCs) [5], and short-chain fatty acids (SCFAs) [6], etc. Among these metabolites, indole not only contributes to halitosis but can also act as a signaling molecule that may influence antibiotic tolerance [7], biofilm formation [8,9], and bacterial virulence [10], potentially contributing to periodontal dysbiosis and disease progression. Oral pathogenic microbes, such as *Porphyromonas*, *Fusobacterium*, and *Prevotella*, can convert tryptophan into indole through tryptophan metabolism and have been implicated in periodontitis and halitosis [9,11,12,13]. However, the bacterial genetic features associated with indole production and their relevance to periodontal pathogens remain unclear.

*Porphyromonas gingivalis* (*P. gingivalis*) is regarded as a key periodontitis pathogen within the red complex (*P. gingivalis*, *Treponema denticola*, and *Tannerella forsythia*) [14,15] and harbors the *tnaA* gene encoding tryptophanase [13]. Tryptophan metabolism is closely linked to *P. gingivalis* virulence [16] and indole production [17]. Mutation of *tnaA* in *P. gingivalis* significantly reduced indole levels, *kgp* expression, and hemin utilization [16], suggesting that *tnaA* may contribute to virulence-associated phenotypes of *P. gingivalis.* Although *P. gingivalis* virulence is influenced by host interactions, environmental conditions, and experimental models, previous studies have provided a broad distinction between strains exhibiting relatively high- or low-virulence phenotypes. In this context, W83, W50, A7436, TDC60, and ATCC 49417 have been frequently described as relatively high-virulence strains, with reported features including higher proteinase activity, stronger invasiveness, and more pronounced periodontal bone destruction, whereas ATCC 33277, AJW4, and 381 have generally been considered relatively low-virulence strains [18,19,20,21,22,23]. In this study, 118 *P. gingivalis* genomes were retrieved from the National Center for Biotechnology Information (NCBI) database. Previous comparative genomic studies have shown that although previously reported high-virulence strains (such as W83) and low-virulence strains (such as ATCC 33277, AJW4) share similar genome sizes and guanine/cytosine (GC) content, they exhibit extensive genomic rearrangements (inversions, translocations, deletions, and substitutions), which may correlate with their virulence differences and endothelial cell invasiveness [19,24]. Moreover, insertion sequences are of particular interest because they have been proposed as a potential contributor to local transcriptional differences in bacteria [25,26,27,28]. Similar insertion sequence-associated changes have been reported in other pathogens, including *Staphylococcus aureus* and *Mycobacterium tuberculosis* [29,30]. Although previous studies have addressed the tryptophan metabolism and genomic diversity of *P. gingivalis* in periodontitis, there is still limited knowledge regarding how genomic polymorphisms and genetic disparities among different strains are associated with tryptophan metabolism.

Here, we investigated the association between indole-related metabolic signatures and periodontitis severity, and compared indole production in W83 and ATCC 33277, two strains previously reported to differ in virulence-associated characteristics. Through comparative genomic analysis, we further explored *tnaA*-adjacent genomic features potentially associated with strain-specific heterogeneity in tryptophan-indole metabolism among *P. gingivalis* strains. By integrating salivary sample analyses, strain-level indole-production assays, and comparative genomics, this work aimed to examine associations between *tnaA*-adjacent genomic features, tryptophan metabolism, and virulence-associated gene expression through multiple methodological approaches.

## 2. Materials and Methods

### 2.1. Culture of Bacterial Strains

*P. gingivalis* W83 and *P. gingivalis* ATCC 33277 were cultured in a modified Brain Heart Infusion Broth medium (BHI; BD Biosciences, Sparks, MD, USA) supplemented with 5 μg/mL hemin and 1 μg/mL vitamin K1 under anaerobic conditions. The bacterial concentration was standardized to an optical density (OD) of 1.0 at 600 nm using a spectrophotometer, corresponding to 1 × 10^9^ bacteria/mL. Blood agar plates for *P. gingivalis* were prepared using the same supplements plus 12 mg/mL agar and 5% defibrillated sheep blood.

For growth curve analysis, overnight cultures of *P. gingivalis* W83 and ATCC 33277 were adjusted to the same optical density and diluted into fresh modified BHI medium to an initial OD600 of 0.2. For indole measurements normalized to bacterial density, cultures were adjusted to OD600 values of 0.1, 0.25, or 1.0 before centrifugation, and equal volumes of the adjusted cultures were centrifuged at 10,000 rpm for 2 min for the Kovács assay. For time-course analysis of indole production and gene expression, W83 and ATCC 33277 cultures were inoculated at the same initial OD600 and incubated anaerobically. At each indicated time point, cultures were centrifuged at 10,000 rpm for 2 min. Supernatants were collected for indole measurement, and the corresponding cell pellets were used for RNA extraction and RT-qPCR analysis. For experiments involving exogenous indole treatment, bacterial cultures were treated with 0.5 mM indole for 24 h before the downstream analyses described in the corresponding sections. Before inoculation and each OD600 measurement, bacterial suspensions were thoroughly mixed to minimize the influence of auto-aggregation. All inoculation procedures were performed under aerobic conditions, and cultures were subsequently incubated under anaerobic conditions.

### 2.2. Kovács Assay

Liquid culture of *P. gingivalis* W83 and *P. gingivalis* ATCC 33277 was harvested at the log phase and diluted to 1 × 10^9^ bacteria/mL, and subjected to different treatments according to the experimental design. The *P. gingivalis* culture was centrifuged (10,000 rpm, 2 min, 4 °C), and the supernatant was transferred into a clean tube. Then, an equal volume of Kovac’s reagent (Solarbio, Beijing, China) was added for reaction at room temperature. A pink to red ring indicated positive indole production, while a yellow layer indicated a negative result. The reaction produced a soluble product, which was measured spectrophotometrically at 530 nm. Standard indole solutions at known concentrations ranging from 0 to 2 mM were analyzed to generate a calibration curve for subsequent quantification.

### 2.3. Reverse Transcription-Quantitative Polymerase Chain Reaction (RT-qPCR) and Reverse Transcription Polymerase Chain Reaction (RT-PCR)

The cultured *P. gingivalis* cells and clinical saliva samples were harvested by centrifugation (10,000 rpm, 2 min, 4 °C) and subsequently treated with lysozyme (0.5 mg/mL) at 37 °C for 30 min. RNA was extracted using AG RNAex Pro RNA Reagent (Accurate Biotechnology Co., Ltd., Changsha, China; AG21102) and converted to cDNA via the Evo M-MLV RT Mix Kit (Accurate Biotechnology Co., Ltd., China; AG11728). RT-qPCR was performed using the Pro Taq HS Premix Probe qPCR Kit (Accurate Biotechnology Co., Ltd., China; AG11730) to quantify relative mRNA levels. For the two-step protocol, genomic DNA was first removed by incubating RNA samples with gDNA Clean Reagent at 42 °C for 2 min, followed by reverse transcription for cDNA synthesis. The RT-qPCR reaction was then performed using the 2 × Pro Taq HS Probe Premix under the following conditions: initial denaturation at 95 °C for 30 s, followed by 40 cycles of denaturation at 95 °C for 5 s and combined annealing/extension at 60 °C for 30 s. For RT-qPCR analyses of cultured *P. gingivalis*, three biological replicates were prepared from independent bacterial cultures for each condition. For RT-qPCR analyses of clinical saliva samples, each biological replicate corresponded to an independent clinical sample. According to previous studies [16], *P. gingivalis*-specific 16S rRNA was used as the bacterial reference gene. The *rgp* primers used in this study specifically targeted *rgpA*, therefore, *rgp* expression refers to *rgpA* expression throughout the manuscript and figures. All primer sequences used in this study are listed in Supplementary Table S6. Relative gene expression levels were calculated using the 2^−ΔΔCt^ method.

For the RT-PCR assay, cDNA synthesized from clinical saliva RNA was used as the template for PCR amplification with *ISPg1*-specific primers (Supplementary Table S6) and Taq DNA polymerase. PCR products were separated by agarose gel electrophoresis to verify the expected amplicon size and confirm amplification.

### 2.4. Human Saliva Sample Collection

Saliva samples were collected in the present study and were used to assess whether *ISPg1* transcripts could be detected and their associations with salivary indole levels, selected bacterial transcripts, and clinical periodontal parameters within a cohort of *P. gingivalis*-positive periodontitis patients. The study was approved by the Ethics Committee of the School of Stomatology, Shandong University (No. 20250901), in accordance with the Declaration of Helsinki. Periodontitis was diagnosed according to the Classification of Periodontal and Peri-Implant Diseases and Conditions outlined in the 2018 World Workshop of Periodontology. Exclusion criteria included systemic diseases, use of antibiotics within the past 6 months, use of non-steroidal anti-inflammatory agents (NSAIDs) or oral contraceptives within the past 3 months, periodontal treatment in the past 12 months, pregnancy, and lactation.

All participants were informed of the purpose of the study before enrollment. Before collection, participants performed oral hygiene procedures and then rinsed their mouths thoroughly with distilled water. Approximately 3 mL of unstimulated whole saliva without coughing up mucus, food debris, or blood was collected from each subject into a sterile graduated tube. The collected samples were placed on ice and transported to the laboratory immediately, and stored at −80 °C. Full-mouth periodontal parameters, including probing depth (PD), clinical attachment loss (CAL), plaque index (PI), calculus index (CI), and gingival index (GI), were examined and recorded by one trained and calibrated practitioner. The RNA extraction and quantification of collected saliva samples are described in detail in Section 2.3.

### 2.5. Metabolomics Data Analysis

A previously reported human salivary metabolomics dataset [16] was used for secondary analysis of indole-related metabolic signatures. The differential metabolites were initially identified using VIP-value > 1 and adjusted *p*-value < 0.01, and then detected metabolites were further filtered using |log_2_FC| > 2 and adjusted *p*-value < 0.01. T Differential metabolites were visualized in volcano plots.

### 2.6. Amplicon Data Sequencing

Our clinical cohort study was approved by the Ethics Committee of Anhui Medical University for Nationalities (No. 20180163) and registered in the Chinese Clinical Trial Registry (ChiCTR1800015652). A total of 218 participants were enrolled in this study, including healthy controls without periodontitis and patients with periodontitis. Total DNA was extracted from subgingival plaque samples using a DNA isolation kit (Qiagen, Germantown, MD, USA; 51704), according to the manufacturer’s protocol. The V3–V4 region of the bacterial 16S rRNA gene was PCR-amplified with primers 338F(5′-GTACTCCTACGGGAGGCAGCA-3′) and 806R(5′-GTGGACTACHVGGGTWTCTAAT-3′), followed by sequencing on the Illumina platform.

### 2.7. 16S rRNA Amplicon Analysis

16S rRNA amplicon data were used to analyze microbial composition and PICRUSt2-predicted functional profiles. Public 16S rRNA amplicon data used in this analysis were obtained from the NCBI SRA database (https://www.ncbi.nlm.nih.gov/sra/, accessed on 5 June 2026) under the BioProject accession number PRJNA863881. Together with data from our clinical cohort, the raw sequencing data were first processed using fastp software (version 0.23.4) [31] to obtain clean reads. Paired-end sequences were then merged using the USEARCH v11 [32] software with the fastq_mergepairs method. After low-quality sequences were filtered with the fastq_filter method, dereplicated reads (fastx_uniques) were denoised using UNOISE3 to generate zero-radius OTUs (zOTUs). Representative sequences were mapped to quality-filtered reads to create an abundance table. Taxonomy was assigned using the SINTAX classifier (cutoff = 0.8) with RDP v19 as the reference database. The resulting zOTU table was used for downstream ecological and statistical analyses. The PICRUSt2 software (version 2.6.2) [33] was used to predict metagenomic functional profiles with default parameters.

### 2.8. Genomes Retrieval, Annotation, and Analysis

Publicly available *P. gingivalis* genomes were used for comparative genomic analysis. All the *P. gingivalis* genomes were downloaded from the NCBI genome database (https://www.ncbi.nlm.nih.gov/datasets/genome/; accessed on 2 September 2025) using the keyword *Porphyromonas gingivalis*. The downloaded nucleotide sequences were annotated by the Bakta software (version 1.9.2) [34] with default parameters. Transposase annotations and classifications were obtained during this step. The annotated amino acid sequences of all 118 genomes and 36 complete genomes were analyzed by the OrthoFinder software (version 3.0.1b1) [35], respectively, to identify orthologous genes and construct a phylogenetic tree based on the homologous genes [36]. The phylogenetic tree was then visualized by the FigTree software (version 1.4.4). Average nucleotide identity (ANI) between whole genomes was computed by the fastANI software (version 1.34) [37]. The annotated faa and gff files of each genome were processed using Roary (version 3.11.2) [38] to perform the pan-genome analysis pipeline. The Count software (version 9.1106 RC1) [39,40] (https://www.iro.umontreal.ca/~csuros/gene_content/count.html, accessed on 9 September 2025) was then used to infer gene-content evolutionary events (gene gain/loss events) using the PGL: propensity for gene loss (Krylov–Wolf–Rogozin–Koonin) method based on the results obtained from the Roary pan-genome analysis. Representative sequences of the representative sequences of genes with gain events were further annotated using the eggnog-mapper tool (https://usegalaxy.eu/) to obtain the COG annotations. 

### 2.9. Alignment Analysis

The multiple sequence alignment (MSA) analysis performed in this study was performed using MAFFT (version 7.407) [41]. Gene-level BLAST analysis against the database was performed using the blast software (version 2.14.1). Combined BLAST searches for multigene modules were performed using the MultiGeneBlast software (Version 3). The gbk files of the upstream and downstream regions of the *tnaA* gene were processed using the R package tidyverse. Nucleotide sequences of the transposase regions within the complete genomes were aligned by the MAFFT software (Version 7.407) using the adjust direction accurately parameter to correct the gene orientation automatically. The resulting alignments were used to construct a neighbor-joining (NJ) phylogenetic tree with default parameters in MEGA 11 software.

### 2.10. Statistical Analysis

Data are presented as the means ± standard deviation of at least three biological replicates. Student’s *t*-test (normally distributed data) or Wilcoxon test (non-normally distributed data) was performed to compare two groups. Multiple-group comparisons were conducted by one-way or two-way analysis of variance (ANOVA). Correlation analysis was performed using Spearman’s method with the R package ggstatsplot (version 0.13.1). *p*-values for multiple testing were adjusted with the Benjamini–Hochberg false discovery rate (FDR) method. Statistical graphs were generated using GraphPad Prism 9.5 or the R package ggplot2 (version 3.5.2). *p* < 0.05 was considered statistically significant.

## 3. Results

### 3.1. Analysis of Indole-Related Metabolic and Microbial Signatures in Periodontitis

**Figure 1:**
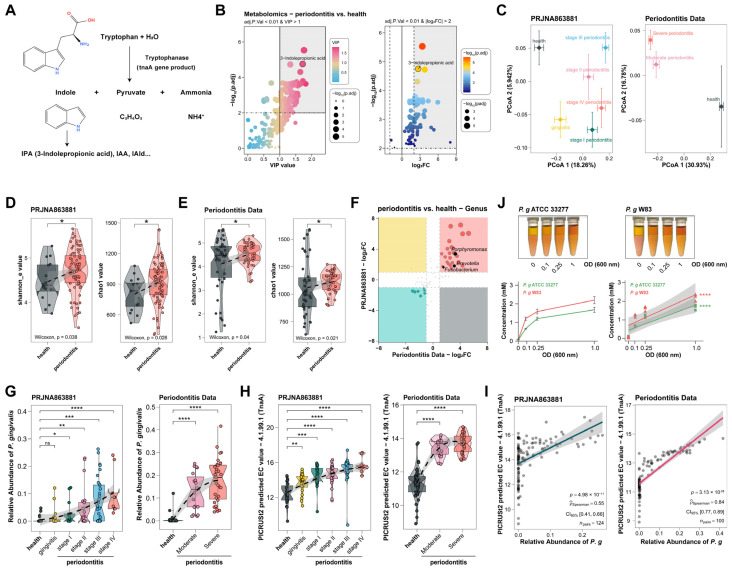
Indole-related metabolic and microbial signatures in periodontitis and indole production by *P. gingivalis* strains. (**A**) Schematic diagram of the bacterial tryptophan-indole metabolic pathway. (**B**) Analysis of differential metabolites in saliva between periodontitis patients and healthy individuals. (**C**) Principal coordinates analysis (PCoA) of the public dataset PRJNA863881 (left) and our clinical periodontitis cohort (right). (**D**,**E**) Alpha diversity (Chao1 index, Shannon_e index) analysis in both cohorts. (**F**) Consistency analysis across both cohorts. Solid black dots represent three genera associated with indole-related metabolism. (**G**) The relative abundance of *P. gingivalis* across different disease severity in both cohorts. (**H**) PICRUSt2-predicted values (log2 transformed) of EC 4.1.99.1 (TnaA) in periodontitis samples across different disease severity. (**I**) Spearman correlation analysis between *P. gingivalis* abundance and EC 4.1.99.1 (TnaA) PICRUSt2-predicted values. Shaded areas: 95% confidence intervals. (**J**) Kovács assay (top), and analysis of the relationship between *P. gingivalis* density and indole concentration (bottom). Colors: red, W83; green, ATCC 33277. Statistical significance: ns, not significant; * *p* < 0.05, ** *p* < 0.01, *** *p* < 0.001, and **** *p* < 0.0001.

As shown in Figure 1A, indole is produced from the degradation of tryptophan by the bacterial tryptophanase and catalyzed by various enzymes to form multiple derivatives, such as 3-indolepropionic acid (IPA), indole acetic acid (IAA), and 3-indole-aldehyde (IAld) [42,43]. To assess whether indole-related metabolic signatures were enriched in periodontitis, we analyzed salivary metabolomics data of periodontitis patients and healthy individuals [16] and identified a total of 50 significantly upregulated metabolites (VIP > 1, adj. *p* < 0.01 and |log_2_FC| > 2) (Figure 1B, Table S1). Of these, IPA, an indole-related metabolite, was one of the most significantly increased metabolites (Figure 1B). Next, we investigated which oral bacteria were associated with indole-related signatures in periodontitis using a public dataset (PRJNA863881) and our clinical periodontitis cohort. Principal coordinate analysis (PCoA) revealed a distinct separation of microbial community structures among healthy controls, gingivitis subjects, and patients with different stages of periodontitis (Figure 1C). The alpha diversity analysis showed that periodontitis patients exhibit significant enrichment of oral microbiome abundance compared to the healthy controls (Figure 1D,E).

Consistency analysis of the differential bacterial genera between the two cohorts revealed 37 genera consistently upregulated across both cohorts (Figure 1F, Table S2), including *Porphyromonas*, *Prevotella*, and *Fusobacterium* that harbor the *tnaA* gene [9,11,12,13]. Among the detected indole-producing genera, *Porphyromonas* exhibited the highest fold change in periodontitis patients compared with healthy controls (Figure 1F). As a representative species of the genus *Porphyromonas,* the relative abundance of *P. gingivalis* was significantly increased in periodontitis patients and exhibited an upward trend with disease severity (Figure 1G). *P. gingivalis* abundance positively correlated with the periodontitis-related clinical parameters, including Plaque Index (PI), Probing Depth (PD), Bleeding on Probing (BOP), and Clinical Attachment Loss (CAL) (Figure S1A).

PICRUSt2-based prediction showed that the log2-transformed values of EC 4.1.99.1 (TnaA) tended to be higher in periodontitis samples (Figure S1D,E) and increased with disease severity (Figure 1H). Correlation analysis based on PICRUSt2-predicted TnaA values revealed a potential positive association with clinical periodontal parameters (PI, PD, BOP, and CAL) (Figure S1F). In addition, correlation analysis indicated a potential positive link between PICRUSt2-derived TnaA predictions and the relative abundance of *P. gingivalis* derived from 16S rRNA data (Figure 1I). In vitro Kovács assay also showed that indole production was positively correlated with the bacterial load of *P. gingivalis* in both ATCC 33277 and W83 strains (Figure S1B,C and Figure 1J). These findings indicate that *P. gingivalis* is enriched in periodontitis and can produce indole under the tested in vitro conditions.

### 3.2. Predicted Functional Profiles and In Vitro Validation of TnaA/Indole-Related Virulence Traits in Two P. gingivalis Strains

**Figure 2:**
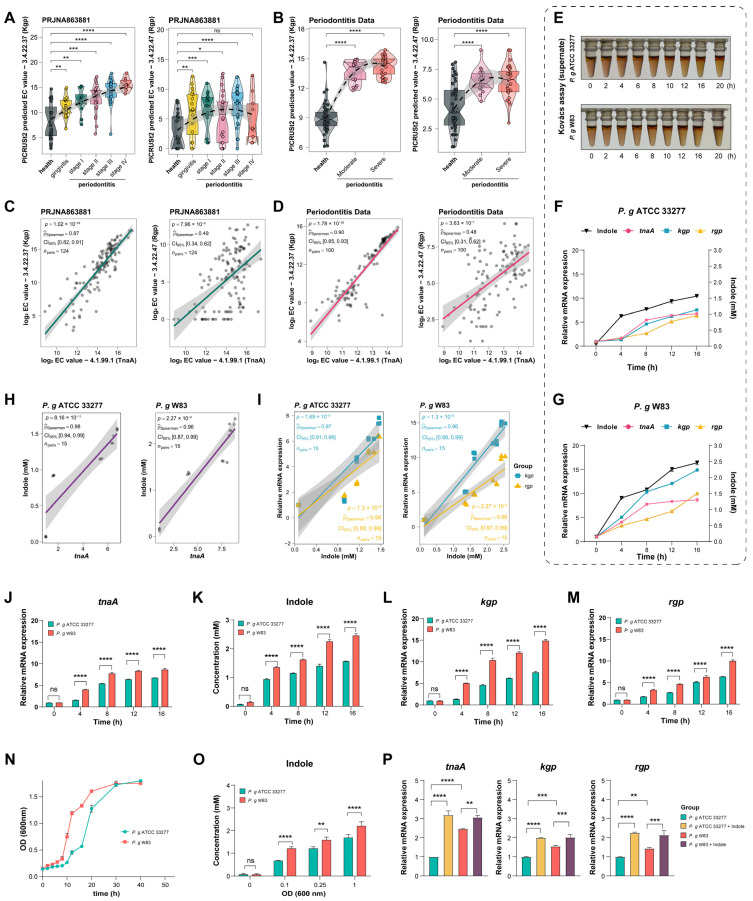
Predicted functional profiles and in vitro validation of TnaA/indole-related virulence traits in two *P. gingivalis* strains. (**A**,**B**) Predicted values calculated by PICRUSt2 (log_2_ transformed) of EC 3.4.22.37 (Kgp) and EC 3.4.22.47 (Rgp) in periodontitis samples across different disease severity. (**C**,**D**) Spearman correlation analysis of TnaA predicted values and Kgp/Rgp predicted values. Shaded areas: 95% confidence intervals. (**E**) Kovács assay for indole production in *P. gingivalis* ATCC 33277 and W83 strains, quantitative results of indole concentrations are detailed in subfigure (**F**,**G**) (black line). (**F**,**G**) The indole concentration and the mRNA expression of *tnaA*, *kgp*, and *rgp* across different time points. The *rgp* signal shown in this figure refers to *rgpA*, based on the primer target used in this study. (**H**,**I**) Spearman correlation analysis between *tnaA* expression and indole concentration, as well as indole concentration and *kgp/rgp* expression in *P. gingivalis* ATCC 33277 and *P. gingivalis* W83 based on F-G. Shaded areas: 95% confidence intervals. (**J**–**M**) Bar charts were used to compare the two strains using the same dataset shown in F–G. (**N**) The growth curve of *P. gingivalis* ATCC 33277 and *P. gingivalis* W83. (**O**) Kovács assay for indole production at equivalent bacterial loads based on OD600 value in both *P. gingivalis* strains. (**P**) The mRNA expression of *tnaA*, *kgp*, and *rgp* after 0.5 mM exogenous indole treatment for 24 h. Statistical significance: ns, not significant; * *p* < 0.05, ** *p* < 0.01, *** *p* < 0.001, and **** *p* < 0.0001.

PICRUSt2 functional inference was conducted to preliminarily predict the potential bacterial functions. The log2-transformed predicted values of EC 3.4.22.37 [Lys-specific gingipain (Kgp)] and EC 3.4.22.47 [Arg-gingipain (Rgp)] were higher in patients with periodontitis (Figure S2A,B) and showed an upward trend with disease severity (Figure 2A,B). Correlation analysis based on predicted Kgp and Rgp values indicated potential positive associations with clinical periodontal parameters (PI, PD, BOP, and CAL) (Figure S2C,D). Spearman analysis further suggested a positive correlation between PICRUSt2-predicted Kgp/Rgp values and TnaA values across the two cohorts (Figure 2C,D).

Kovács assays were performed to evaluate indole production; semi-quantification was performed as described in Materials and Methods. RT-qPCR was used to quantify the expression of *kgp* and *rgp* (*rgpA* in this study) at various time points in *P. gingivalis* ATCC 33277 and W83. The results showed that indole concentration and *tnaA* expression were positively correlated with *kgp* and *rgp* expression in both strains (Figure 2E–I and Figure S2E). Of note, line graphs were used to show the temporal changes of indole abundance and gene expression in each strain, whereas bar charts were used to compare the two strains at each time point using the same dataset shown in Figure 2F,G. We found that *tnaA* expression and indole abundance were consistently higher in W83 and lower in ATCC 33277 across all examined time points (Figure 2J,K). Similarly, the mRNA levels of *kgp* and *rgp* showed the same trends at all time points (Figure 2L,M). OD600-based growth monitoring showed that W83 reached a higher optical density earlier than ATCC 33277 under the tested culture conditions (Figure 2N, Figure S3A). We further analyzed indole production at matched OD600 values. Consistent with the line-graph analysis in Figure 1J, the bar-chart comparison showed that indole levels were significantly higher in W83 than in ATCC 33277 (Figure 2O). However, because the two strains exhibited different growth kinetics, these transcriptional differences may partly reflect distinct physiological or growth states at the sampled time points rather than growth-independent strain-specific regulation.

The ability of gingipains to cleave hemoglobin may provide *P. gingivalis* with a usable source of heme for growth [44,45] and is associated with colonies on blood agar producing black pigment [29]. After anaerobic incubation on blood agar plates, both strains formed black-pigmented colonies, but W83 developed black pigmentation earlier than ATCC 33277 (Figure S3B,C). To test whether indole could influence the expression of *P. gingivalis* virulence-associated genes, 0.5 mM exogenous indole was added to the culture medium for 24 h. The results showed that exogenous indole significantly upregulated the mRNA expression of *tnaA*, *kgp*, and *rgp* in both *P. gingivalis* W83 and *P. gingivalis* ATCC 33277 (Figure 2P). Combining PICRUSt2 functional predictions and in vitro assays using two *P. gingivalis* strains, these results suggest that TnaA and indole may be associated with virulence-associated features between the tested strains.

### 3.3. Amino Acid Sequences of TnaA Are Highly Conserved Across P. gingivalis Strains with Different Reported Virulence Backgrounds

**Figure 3:**
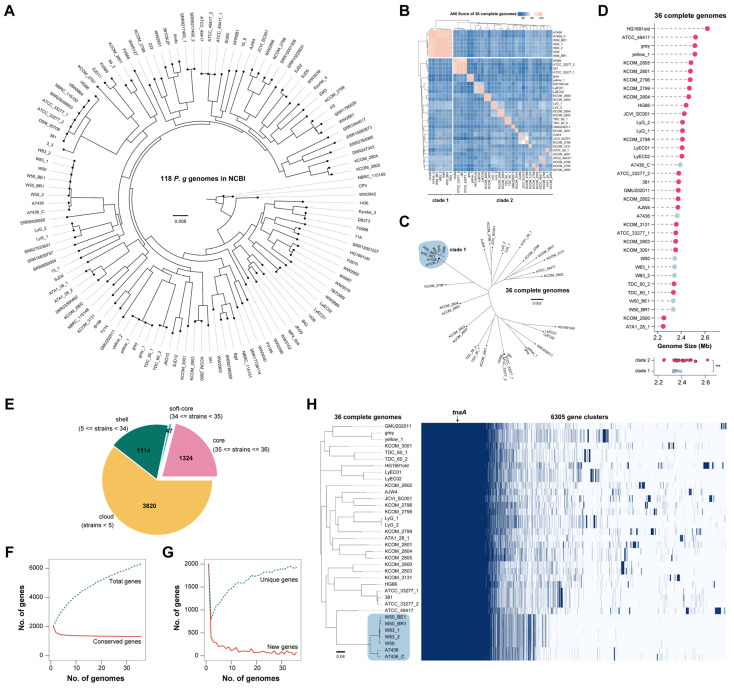
Phylogenetic tree and pan-genome analysis of 36 *P. gingivalis* strains. (**A**) Phylogenetic tree of 118 *P. gingivalis* genomes based on all the orthologous genes calculated by the OrthoFinder software. (**B**) ANI analysis of 36 *P. gingivalis* strains with a complete genome. (**C**) The Maximum Likelihood (ML) phylogenetic tree of 36 *P. gingivalis* strains with a complete genome based on the single-copy orthologous genes. (**D**) Distribution and statistical comparison of genome sizes among 36 strains. ** *p* < 0.01. (**E**) Number of core genes, soft-core genes, shell genes, and cloud genes from the pan-genome analysis. (**F**) Curves of conserved and total genes based on the pan-genome analysis. (**G**) Curves of new and unique genes based on the pan-genome analysis. (**H**) The distribution of gene families from pan-genome analysis among 36 strains by Roary software. The gene matrix shows the presence in blue and absence in white. The dendrogram in the phylogenetic relationship of 36 *P. gingivalis* strains based on the pan-genome analysis.

We obtained the genomic data of 118 *P. gingivalis* strains from the NCBI database, including 36 strains with complete genome sequences and 82 with draft sequences (Table S3). All the downloaded nucleotide sequences were annotated by the Bakta software, and the annotated amino acid sequences were analyzed by the OrthoFinder software to obtain all orthologous genes. The statistical information of all downloaded *P. gingivalis* strains is presented in Table 1 (complete genomes). The phylogenetic tree based on all the orthologous genes of 118 *P. gingivalis* strains by OrthoFinder is shown in Figure 3A. In addition, average nucleotide identity (ANI) analysis and phylogenetic analysis of 36 strains with complete genomes showed that the previously reported high-virulence strains such as W83, W50, and A7436 clustered together on one branch with a higher intra-clade similarity (defined as clade 1), whereas the remaining strains were distributed in another branch (defined as clade 2) (Figure 3B,C), and previous studies on the virulence heterogeneity among different *P. gingivalis* strains were summarized in Table S4. Genome size analysis showed that the previously reported high-virulence strains (strains in clade 1, TDC_60_1 and TDC_60_2, were also reported as high-virulence strains [23]) possessed comparatively small genomes compared to others (strains in clade 2) (Figure 3D).

**Figure 4:**
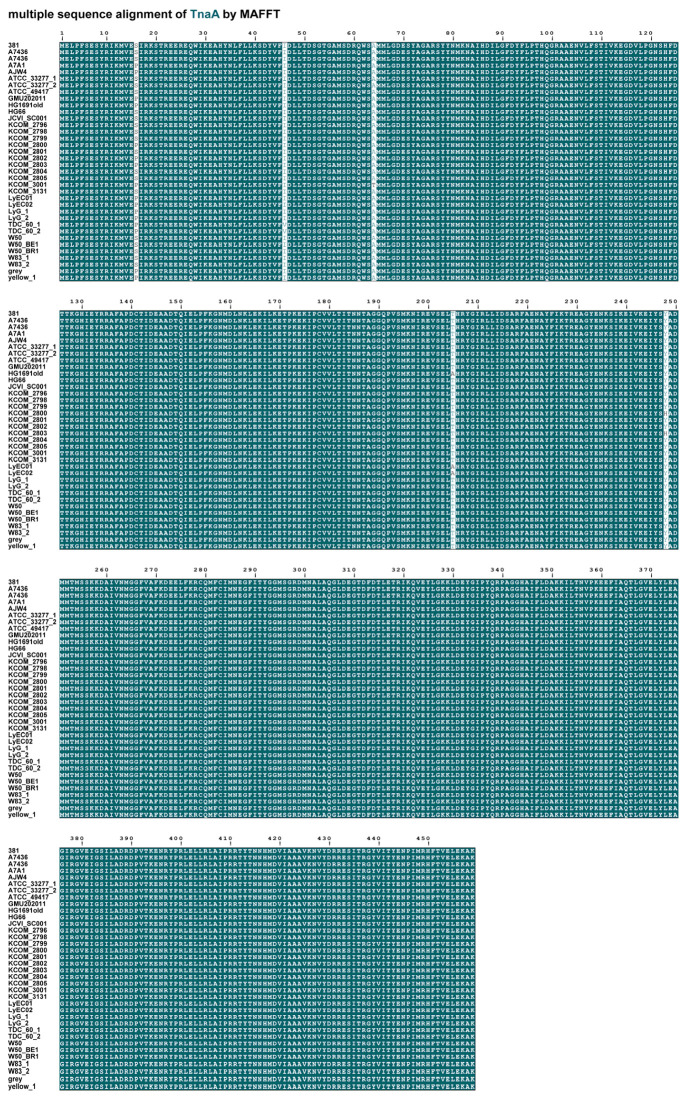
Multiple sequence alignment (MSA) results revealed highly conserved amino acid sites in the TnaA across *P. gingivalis* strains.

Pangenomic analysis showed, regarding the gene frequency, 1324 core genes (35 ≤ strains ≤ 36), 47 soft-core genes (34 ≤ strains < 35), 1114 shell genes (5 ≤ strains < 34), and 3820 cloud genes (strains < 5) were identified across the 36 strains with a complete genome (Figure 3E). Both the total and unique gene numbers continued to increase with the addition of new genomes without reaching an asymptotic behavior, indicating that the pan-genome of *P. gingivalis* remains open (Figure 3F,G). We assessed the gene distribution patterns across the strains and found that *tnaA* was shared by almost all the *P. gingivalis* strains, belonging to a single-copy orthologous gene (Figure 3H). MSA of TnaA among different strains also revealed highly conserved amino acid sequences (Figure 4), suggesting limited amino acid-sequence divergence in TnaA among the analyzed strains, prompting us to further examine strain-associated genomic variation in the *tnaA*-flanking region.

### 3.4. Variation of the Transposase Domain Adjacent to TnaA in P. gingivalis Strains of Different Virulence

**Figure 5:**
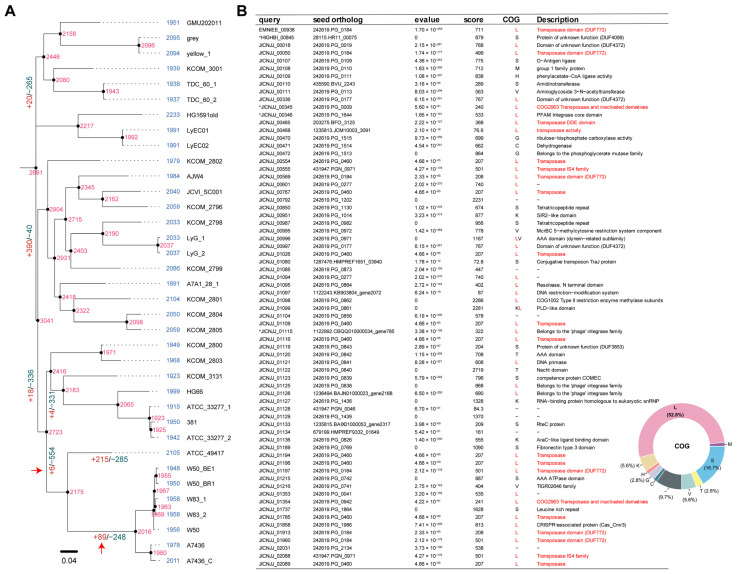
Gene gain/loss event analysis and gene annotations across *P. gingivalis* strains. (**A**) The results of the *P. gingivalis* phylogenetic tree (based on the pan-genome analysis) and gene gain/loss event analysis using Count software. The number of gene loss events (green) and gene gain events (red) was shown on the branch. (**B**) The results of gene annotation analysis of genes with gain events by eggnog-mapper. The pie chart shows category L accounts for 52.8%, representing the most abundant category. Query with * stands for the reference sequence from the +6 gene gain event.

To explore genomic features potentially associated with strain-specific indole-related phenotypes, we analyzed the *tnaA*-adjacent transposase region. Based on the *P. gingivalis* phylogenetic tree constructed based on pan-genome analysis, combined with the gene gain/loss event analysis by Count software, we found that the previously reported high-virulence strains (W83, W50, A7436) exhibited more gene loss events across evolutionary branches (green numbers) (Figure 5A, Table S5), consistent with the smaller genome size observed in Figure 3D. Specifically, the previously reported high-virulence strain branch (W83, W50, A7436) gained 95 genes (6 plus 89 as indicated by the red arrow) during the evolutionary phase compared to others (Figure 5A). We performed gene annotation analysis of the 95 acquired genes by eggnog-mapper, and a total of 70 genes were successfully annotated (Figure 5B, Table S7). Most of these genes (52.8%) were classified into COG functional category L, which is primarily associated with DNA replication, recombination, and repair. Protein functional annotation revealed that these genes are predominantly functionally enriched in transposase-related biological processes (Figure 5B). These results suggested that previously reported high-virulence strains may have acquired more transposase-associated genes during evolution.

**Figure 6:**
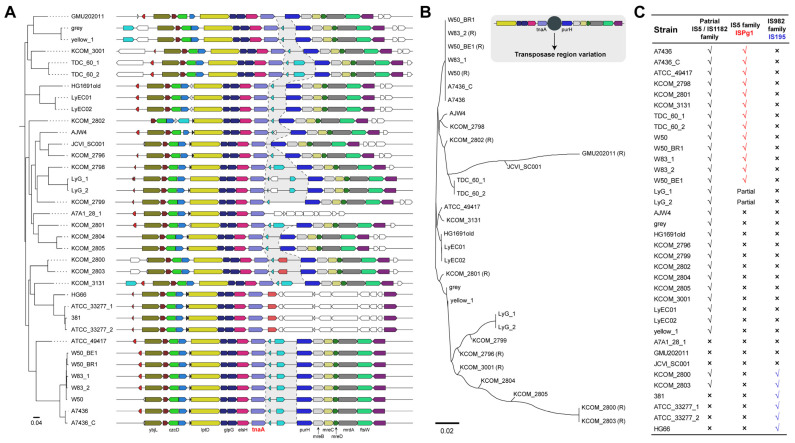
Genomic characteristics and distribution of the *tnaA*-associated transposase region in *P. gingivalis* strains. (**A**) MultiGeneBlast analysis of *tnaA* and its neighboring genomic regions (10 upstream and downstream genes around *tnaA*) among 36 *P. gingivalis* strains with a complete genome. The left panel shows the strain clustering tree; the middle-colored blocks represent genes with different functions (gene names are labeled at the bottom). (**B**) Neighbor-Joining (NJ) phylogenetic tree of *P. gingivalis* strains constructed from nucleotide sequences between *tnaA* and *purH* (transposase region). (**C**) Distribution profile of the transposase region across 36 *P. gingivalis* strains. “√” indicates presence and “×” indicates absence.

Next, we extracted the genomic regions of 10 genes upstream and downstream of *tnaA* from 36 *P. gingivalis* strains with complete genome sequences. Based on MultiGeneBlast analysis, the genes within these regions were blasted against each other, and the orientation and arrangement were aligned and visualized (Figure 6A). The genes upstream of *tnaA* and downstream of *purH* showed conserved gene orientation and arrangement across *P. gingivalis* strains. Notably, distinct gene fragments were identified within this region (Figure 6A) and were subsequently annotated as transposase-associated region (Figure 6B). Previously reported high-virulence strains, including W83, W50, and A7436, shared a highly similar transposase region. Furthermore, we performed MSA of the transposase region based on the nucleotide sequences, followed by the construction of a neighbor-joining (NJ) phylogenetic tree (Figure 6B). The previously reported high-virulence strains W83, W50, and A7436 clustered within the same branch. These findings suggest that the transposase regions share common evolutionary characteristics in previously reported high-virulence strains compared to other strains.

Gene annotation of the transposase regions revealed that the previously reported high-virulence strains W83, W50, A7436, TDC60, and ATCC 49417 harbor the IS5 family ISPg1 transposase, whereas the previously reported low-virulence strains ATCC 33277, 381, and HG66 carry the IS982 family IS195 transposase (Figure 6C). These results indicate that variation in the *tnaA*-adjacent transposase region varies among *P. gingivalis* strains with different previously reported virulence-related backgrounds. However, the relationship between this genomic variation and experimentally assessed virulence phenotypes requires further validation.

### 3.5. Detection and Correlation Analysis of ISPg1 and Related Virulence Genes in Periodontitis Saliva Samples

**Figure 7:**
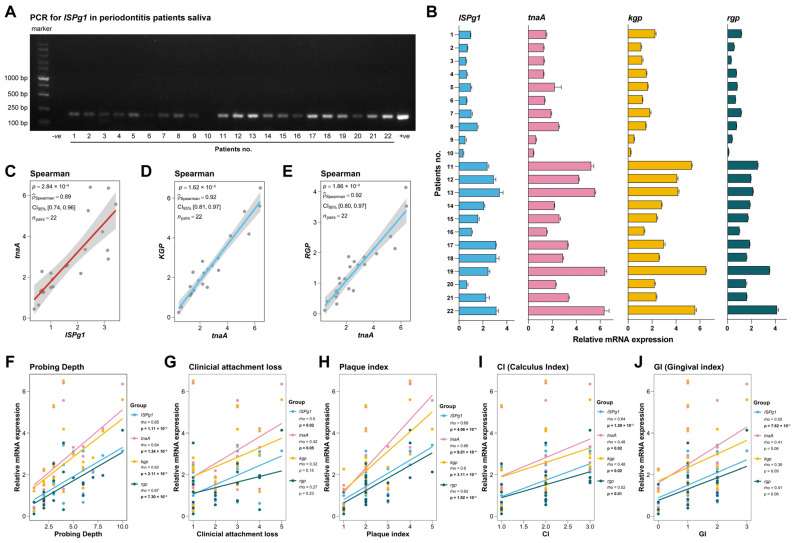
Detection and correlation analysis of *ISPg1* and related virulence genes in periodontitis samples. (**A**) RT-PCR detection of *ISPg1* in saliva samples from periodontitis patients. “-ve”: negative control; “+ve”: positive control; 1–22: patient numbers. (**B**) RT-qPCR analysis showed relative mRNA expression levels of ISPg1, *tnaA*, *kgp*, and *rgp* in 22 saliva samples after normalization to *P. gingivalis*-specific 16S rRNA. (**C**–**E**) Spearman correlation analysis: (**C**), Correlation between *ISPg1* and *tnaA* expression; (**D**,**E**), Correlation between *tnaA* and *kgp/rgp* expression. Shaded areas indicate the 95% confidence intervals of the fitted regression lines. (**F**–**J**) Associations between mRNA expression levels of *ISPg1*, *tnaA*, *kgp*, *rgp*, and clinical periodontal parameters: probing depth (PD), clinical attachment loss (CAL), plaque index (PI), calculus index (CI), and gingival index (GI).

To explore whether *ISPg1* could be detected in clinical periodontitis samples, we analyzed saliva samples from 22 patients with periodontitis (Supplementary Table S8, Figure 7A). *ISPg1* transcripts were detectable in most included saliva samples. RT-qPCR was used to assess *ISPg1*, *tnaA*, *kgp*, and *rgp* transcript levels in this patient cohort after normalization to *P. gingivalis*-specific 16S rRNA (Figure 7B). Spearman correlation analysis showed that *ISPg1* transcript levels were positively associated with *tnaA* transcript levels (Figure 7C). As a key gene involved in tryptophan-indole metabolism, *tnaA* transcript levels were positively associated with *kgp* and *rgp* transcript levels (Figure 7D,E). Within this cohort of periodontitis patients, *ISPg1* and *tnaA* transcript levels were positively associated with PD, CAL, PI, CI, and GI with variable correlation strengths (Figure 7F–J). For *kgp* and *rgp*, two virulence-associated genes, transcript levels were positively associated with PD, PI, and CI, whereas associations with CAL were not statistically significant.

Ethylenediaminetetraacetic acid (EDTA) was used as an exploratory perturbation because it can affect bacterial metal homeostasis and cellular stress pathways [46], and because stress conditions have been reported to affect transposase-associated transcriptional responses [47]. The experimental results showed that EDTA treatment was associated with coordinated decreases of *ISPg1*, *tnaA*, *kgp*, and *rgp* expression and indole levels. However, because EDTA induces broad physiological effects, these findings do not establish a functional role for *ISPg1* or *ISPg1*-specific regulation of *tnaA* and should be interpreted only as exploratory observations (Figure S4).

## 4. Discussion

Indole and its derivatives are increasingly recognized as multifunctional bacterial metabolites that regulate microbial community behavior and host responses [48,49,50,51]. Previous studies have mainly focused on the gastrointestinal tract, where indole modulates mucosal immunity and gut permeability via the AHR pathway [52,53,54]. However, its roles in the oral cavity, particularly in periodontitis, have remained largely unexplored. Our findings suggest that indole-related metabolic signatures may represent a metabolic feature associated with microbial dysbiosis and periodontitis.

A previous study reported that disruption of *tnaA* markedly reduced indole accumulation and downregulated gingipain Kgp expression [16], suggesting a connection between tryptophan metabolism and virulence-associated phenotypes. Consistent with this concept, in the two strains examined here, W83 showed higher accumulated indole levels than ATCC 33277 under matched bacterial-density conditions, accompanied by higher expression of *tnaA*, *kgp*, and *rgp*. Because W83 and ATCC 33277 exhibited different growth kinetics, the time-matched transcriptional comparison may reflect distinct physiological states rather than strain-specific regulatory differences. In addition, exogenous indole enhanced the expression of these virulence-associated genes in both strains. Notably, a previous study reported increased *rgp* expression in a *tnaA*-deletion mutant [16]. One possible explanation for this apparent discrepancy is that *tnaA* deletion represents a stable genetic perturbation that may induce metabolic remodeling or compensatory responses, whereas exogenous indole treatment reflects short-term exposure to an indole signal. Microenvironmental signals have also been reported to modulate *P. gingivalis* virulence-associated behaviors; for example, cortisol promotes surface translocation and increases the expression of T9SS-associated genes in *P. gingivalis*, supporting the idea that environmental or metabolic cues may affect pathogenic traits in *P. gingivalis* [55]. Gingipains are major cysteine proteases involved in hemoglobin degradation, nutrient acquisition, black-pigmented colony formation, and periodontal tissue destruction [44,56]. The previously reported high-virulence strains ATCC 49417 and W50 showed faster colony pigmentation than the low-virulence reference strains ATCC 33277 and 381 [20]. Saliva-based studies have further linked total protease activity and *P. gingivalis* gingipain activity to periodontal inflammatory status and clinical parameters [57].

In the two strains examined here, W83 showed higher indole accumulation than ATCC 33277 under the tested conditions. MSA of TnaA across strains revealed highly conserved amino acid sequences. However, conservation of the coding sequence does not exclude potential differences in transcriptional regulation, post-transcriptional processes, protein abundance, or enzymatic activity. Our genomic analysis indicated that variation in the *tnaA*-*purH* transposase-associated region was associated with strain-specific indole-related features. However, whether this local genomic variation directly affects *tnaA* expression remains unresolved, and further functional analyses will be required to determine whether local transcriptional activity is affected. Genomic plasticity has long been recognized as a hallmark of *P. gingivalis* evolution [19]. Comparative genomic analyses have revealed extensive genome rearrangements, reflecting adaptation to changing host niches [19,58]. Genome-based bioinformatic reconstruction has previously been used to predict lipopolysaccharide biosynthetic pathways in *P. gingivalis* W50 and to compare inferred pathways across completely sequenced pathogenic strains, supporting the utility of comparative genomic approaches for identifying strain-level biosynthetic and virulence-associated features in *P. gingivalis* [59]. We found that previously reported high-virulence lineages possessed more transposon-related gene gains, especially in the COG category L, which is associated with DNA replication, recombination, and repair. Although insertion sequences in other bacteria can influence nearby gene expression [60,61], these mechanisms were not directly tested in the present study. Therefore, the ISPg1-containing *tnaA*–*purH* region should be interpreted as a candidate genomic feature associated with *tnaA* expression, rather than evidence of direct regulation. Similar insertion sequence-associated changes have been reported in other pathogens, including *Staphylococcus aureus* and *Mycobacterium tuberculosis* [29,30], providing a biological rationale for this hypothesis. However, these observations do not establish that *ISPg1* exerts a comparable regulatory function in *P. gingivalis*. These observations generate a testable hypothesis that mobile genetic elements may be associated with metabolic and virulence-associated heterogeneity among *P. gingivalis* strains for future studies.

We observed associations between salivary *ISPg1* expression and clinical periodontal parameters, including PD, CAL, and PI. However, the limited sample size, absence of healthy controls, and observational nature of this cohort preclude conclusions regarding disease specificity or causal relationships. Although the saliva-based transcript levels were normalized to *P. gingivalis*-specific 16S rRNA, this normalization cannot fully exclude the possibility that the detected *ISPg1* transcript signal may partially reflect differences in the abundance or composition of *ISPg1*-containing strains rather than transcriptional regulation alone. Our perturbation experiments further showed coordinated changes among *ISPg1* expression, *tnaA* expression, and indole production, but they do not demonstrate *ISPg1*-specific regulation. Together, these findings suggest that strain-specific mobile genetic elements, particularly ISPg1-containing regions, may be associated with tryptophan-indole metabolism and virulence-associated gene expression in *P. gingivalis*.

Although this study integrates comparative genomics, strain-level phenotypic assays, and exploratory clinical sample analysis to identify a transposase-associated genomic feature linked to tryptophan-indole metabolism in *P. gingivalis*, several limitations remain to be addressed in future work. The current study is associative and does not demonstrate direct causality between the *ISPg1*-containing region and *tnaA* transcription, indole production, or virulence-associated phenotypes. Future studies involving *ISPg1* deletion or replacement, genetic complementation, promoter mapping, reporter assays, transcriptional start-site mapping, and transcriptomic analyses are required. Functional analyses were performed only in W83 and ATCC 33277; therefore, the observed differences require confirmation in additional genetically characterized strains and clinical isolates before being extended to broader *P. gingivalis* lineages. The relatively small clinical cohort without healthy controls limits the interpretation of the disease specificity and clinical relevance of the salivary ISPg1-associated findings. PICRUSt2-based functional inference reflects predicted metabolic potential rather than direct metabolic measurements. Further metagenomic, metatranscriptomic, or functional analyses will be required to evaluate whether these predicted functional differences are reflected at the molecular or phenotypic levels.

## 5. Conclusions

In summary, *P. gingivalis* is one of the oral bacterial species capable of indole production. Under the tested conditions, *P. gingivalis* W83 exhibited a greater indole-producing capacity than ATCC 33277. This strain-specific difference is unlikely to be explained by variation in the TnaA amino acid sequence alone, as TnaA was highly conserved across *P. gingivalis* genomes. Instead, genomic variation in the transposase region adjacent to *tnaA*, particularly the presence of *ISPg1*, may be associated with tryptophan-indole metabolism and *P. gingivalis* strain-level virulence-associated features. The positive associations with periodontal clinical parameters were also observed in our periodontitis saliva cohort, but direct causality or *ISPg1*-specific regulation requires further investigation.

## Figures and Tables

**Table 1:** The genome statistics and annotation summary of 36 *P. gingivalis* strains.

	Max	Min	Ave
Genome size (bp)	2,621,167	2,249,024	2,400,032
Contigs/replicons	1	1	1
GC	48.60%	47.90%	48.29%
N50 (bp)	2,621,167	2,249,024	2,400,032
N90 (bp)	2,621,167	2,249,024	2,400,032
N ratio	1.20%	0.00%	0.03%
coding density	87.40%	85.90%	87.02%
tRNAs	54	44	53
tmRNAs	1	1	1
rRNAs	12	3	11.6
ncRNAs	5	3	4
ncRNA regions	9	8	8
CRISPR arrays	6	2	4
CDSs	2259	1882	2036
hypotheticals	290	169	202
pseudogenes	29	8	17

## Data Availability

The public periodontitis data used in this study could be searched and obtained from the NCBI database under the project accession number PRJNA863881. The raw sequence data from our own clinical samples reported in this paper have been deposited in the Genome Sequence Archive [62] in the National Genomics Data Center [63], China National Center for Bioinformation/Beijing Institute of Genomics, Chinese Academy of Sciences (GSA: CRA022445), which are publicly accessible at https://ngdc.cncb.ac.cn/gsa (accessed on 5 June 2026).

## Supplementary Materials

The following supporting information can be downloaded at: https://www.mdpi.com/article/10.3390/pathogens15060617/s1, Figure S1: (A) Spearman correlation analysis of *P. gingivalis* abundance and clinical periodontal parameters: plaque index (PI), probing depth (PD), bleeding on probing (BOP), and clinical attachment loss (CAL). (B,C) Kovács assay for standard indole solutions at different concentration gradients (0–2 mM) was prepared in ddH_2_O and BHI medium. (D,E) Predicted values calculated by PICRUSt2 (log2 transformed) of EC 4.1.99.1 (TnaA) in the healthy group and periodontitis group. (F) Spearman correlation analysis of TnaA predicted values and periodontal clinical indicators (PI, PD, BOP, CAL). Statistical significance: ns, not significant; * *p* < 0.05, ** *p* < 0.01, *** *p* < 0.001, and **** *p* < 0.0001; Figure S2: (A,B) Predicted values calculated by PICRUSt2 (log_2_ transformed) of EC 3.4.22.37 (Kgp) and EC 3.4.22.47 (Rgp) in the healthy group and periodontitis group. (C,D) Spearman correlation analysis between Kgp/Rgp predicted values and clinical periodontal parameters (PI, PD, BOP, CAL). (E) Spearman correlation analysis of *tnaA* expression and *kgp*/*rgp* expression levels in both *P. gingivalis* ATCC33277 and *P. gingivalis* W83 strains. The *rgp* primers specifically targeted *rgpA*. Statistical significance: ns, not significant; * *p* < 0.05, ** *p* < 0.01, *** *p* < 0.001, and **** *p* < 0.0001; Figure S3: (A) Comparison of growth between *P. gingivalis* ATCC 33277 and W83 at 0, 2, 4, 6, 8, 10, 12, 16, 20, 30, and 40 h. (B) Representative images of colony pigmentation on blood agar plates after 96–144 h of anaerobic incubation. (C) Quantitative analysis of Figure S3B. Statistical significance: ns, not significant; * *p* < 0.05, ** *p* < 0.01, *** *p* < 0.001, and **** *p* < 0.0001; Figure S4: (A) Effect of EDTA concentrations on *P. gingivalis* growth. The concentration of 0.5 mM was chosen for the subsequent experiments. (B,C) Relative mRNA expression of *ISPg1* and *tnaA*. (D) Kovács assay (left) and quantitative detection (right). (E,F) Relative mRNA expression of *kgp* and *rgp*. (G–J) Spearman correlation analysis between *ISPg1* and *tnaA*, *tnaA* and indole, *ISPg1* and indole, indole and *kgp*/*rgp*. Statistical significance: ns, not significant; * *p* < 0.05, ** *p* < 0.01, *** *p* < 0.001, and **** *p* < 0.0001; Table S1: Significantly upregulated salivary metabolites between periodontitis patients and healthy individuals; Table S2: Differential bacterial genera consistently upregulated in periodontitis across two cohorts; Table S3: Summary of *P. gingivalis* genomes retrieved from the NCBI database; Table S4: Previously reported virulence-related characteristics of selected *P. gingivalis* strains; Table S5: Gene gain/loss events inferred across *P. gingivalis* evolutionary branches; Table S6: Primer sequences used for RT-qPCR and RT-PCR; Table S7: eggNOG-mapper annotation of 70 successfully annotated genes from the 95 gained genes; Table S8: Clinical information and periodontal parameters of patients included in the saliva cohort.
