# Supplementary material for: Transposase-Associated Variation near tnaA in Porphyromonas gingivalis Is Linked to Indole Production and Virulence-Associated Gene Expression
Source: Pathogens. 2026 Jun 9;15(6):617. doi: 10.3390/pathogens15060617 (PMC13305171; doi:10.3390/pathogens15060617)
Supplement: Supplementary file 1 [file pathogens-15-00617-s001.zip › Supplementary information.pdf]

Supplementary information

Transposase-associated variation near *tnaA* in *Porphyromonas gingivalis* is linked to indole production and virulence-associated gene expression

Li Wei [1], Chengjia Xie [1], Qingnan Ren [2], Mengfan Zhi [1], Song Shen [1], Xiufeng Gu [1], Qiang Feng [1,3,*] and Tianyong Sun [1,*]

[1] Department of Human Microbiome, School and Hospital of Stomatology, Cheeloo College of Medicine, Shandong University & Shandong Key Laboratory of Oral Diseases & Shandong Engineering Research Center of Dental Materials and Oral Tissue Regeneration & Shandong Provincial Clinical Research Center for Oral Diseases, Jinan 250012, China; weilimail@mail.sdu.edu.cn (L.W.); xiecj_cherry@126.com (C.X.); dream-zmf@outlook.com (M.Z.); shensong@sdu.edu.cn (S.S.); 202062070617@sdu.edu.cn (X.G.)

[2] Department of Vascular Surgery, General Surgery, Qilu Hospital of Shandong University, Jinan 250012, China; 202315578@mail.sdu.edu.cn

[3] Shandong University-BOP Joint Oral Microbiome Laboratory, Jinan 250012, China

* Correspondence: fengqiang@sdu.edu.cn (Q.F.); sty@sdu.edu.cn (T.S.); Fax: +86-53188382923 (Q.F. & T.S.)

This file includes:

Figures S1-S4

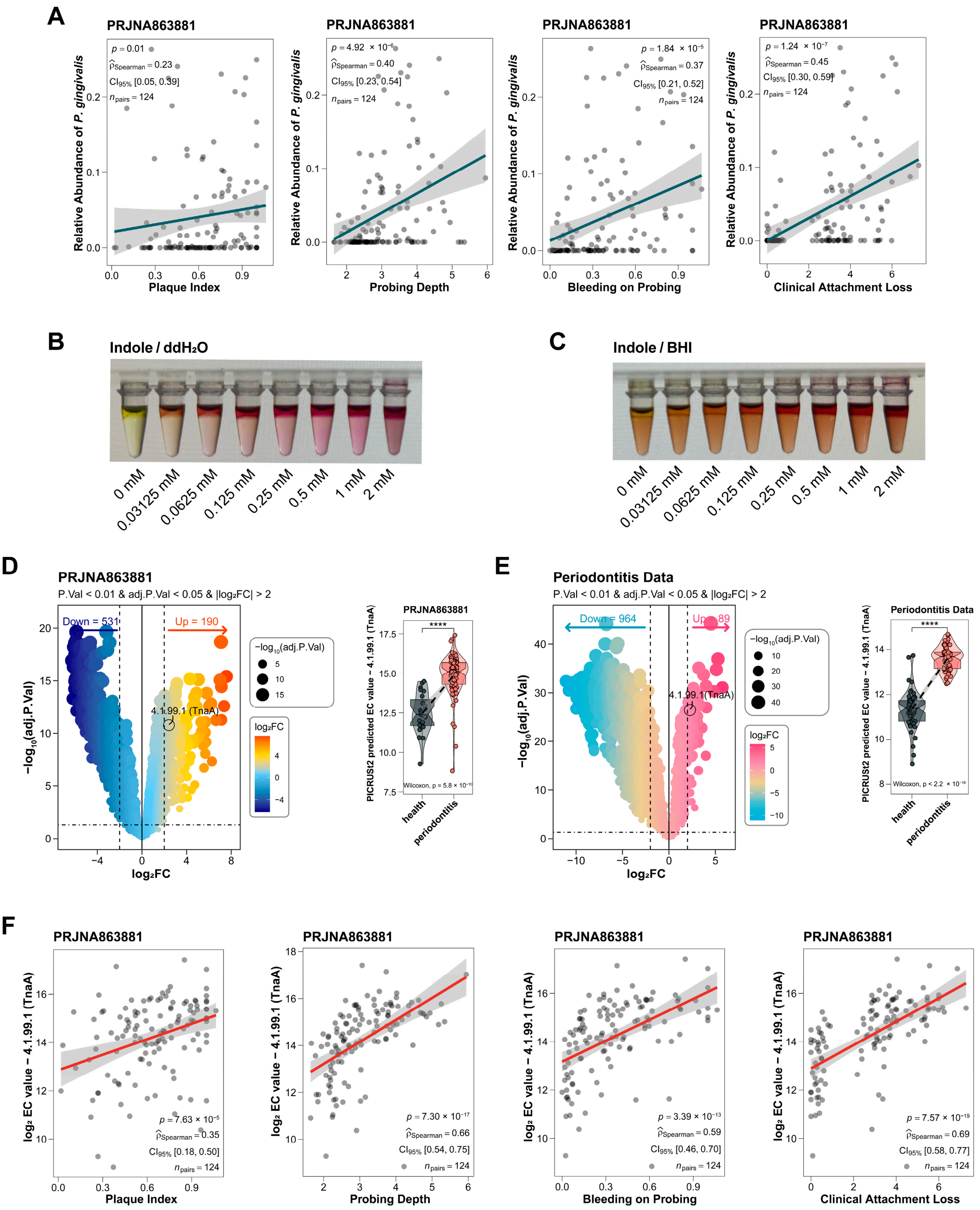

Fig. S1 (**A**) Spearman correlation analysis of *P. gingivalis* abundance and clinical periodontal parameters: plaque index (PI), probing depth (PD), bleeding on probing (BOP), and clinical attachment loss (CAL). Shaded areas: 95% confidence intervals. (**B,C**) Kovács assay for standard indole solutions at different concentration gradients (0-2 mM) was prepared in ddH$_2$O and BHI medium. (**D,E**) Predicted values calculated by PICRUSt2 (log2 transformed) of EC 4.1.99.1 (TnaA) in the healthy group and periodontitis group. (**F**) Spear-man correlation analysis of TnaA predicted values and periodontal clinical indicators (PI, PD, BOP, CAL). Shaded areas: 95% confidence intervals. Statistical significance: ns, not significant; $*P < 0.05$, $**P < 0.01$, $***P < 0.001$, and $****P < 0.0001$.

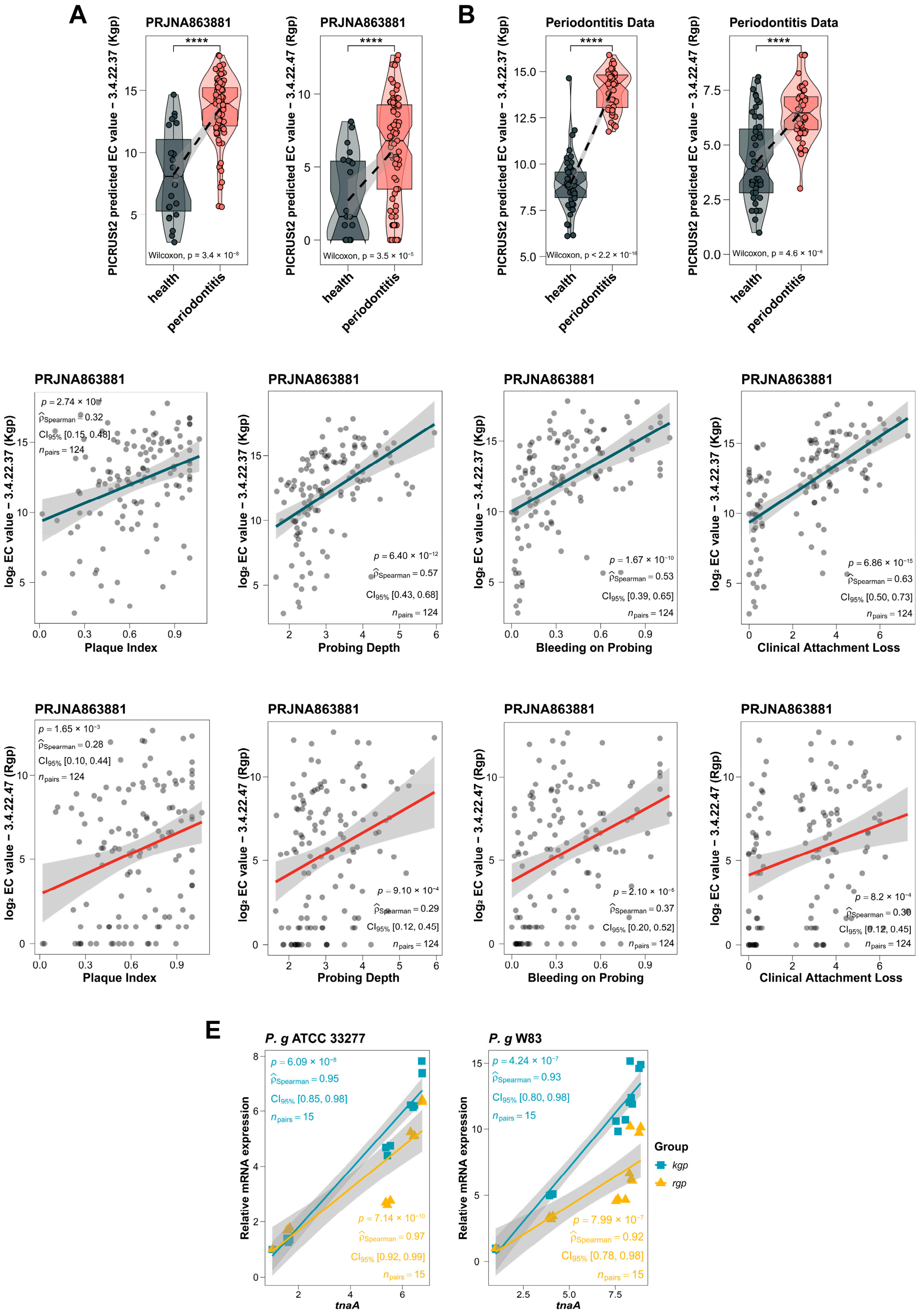

Fig. S2 (**A,B**) Predicted values calculated by PICRUSt2 ($\log_2$ transformed) of EC 3.4.22.37 (Kgp) and EC 3.4.22.47 (Rgp) in the healthy group and periodontitis group. (**C,D**) Spearman correlation analysis between Kgp/Rgp predicted values and clinical periodontal parameters (PI, PD, BOP, CAL). (**E**) Spearman correlation analysis of *tnaA* expression and *kgp*/*rgp* expression levels in both *P. gingivalis* ATCC33277 and *P. gingivalis* W83 strains. The *rgp* primers specifically targeted *rgpA*. Shaded areas: 95% confidence intervals. Statistical significance: ns, not significant; *$P < 0.05$, **$P < 0.01$, ***$P < 0.001$, and ****$P < 0.0001$.

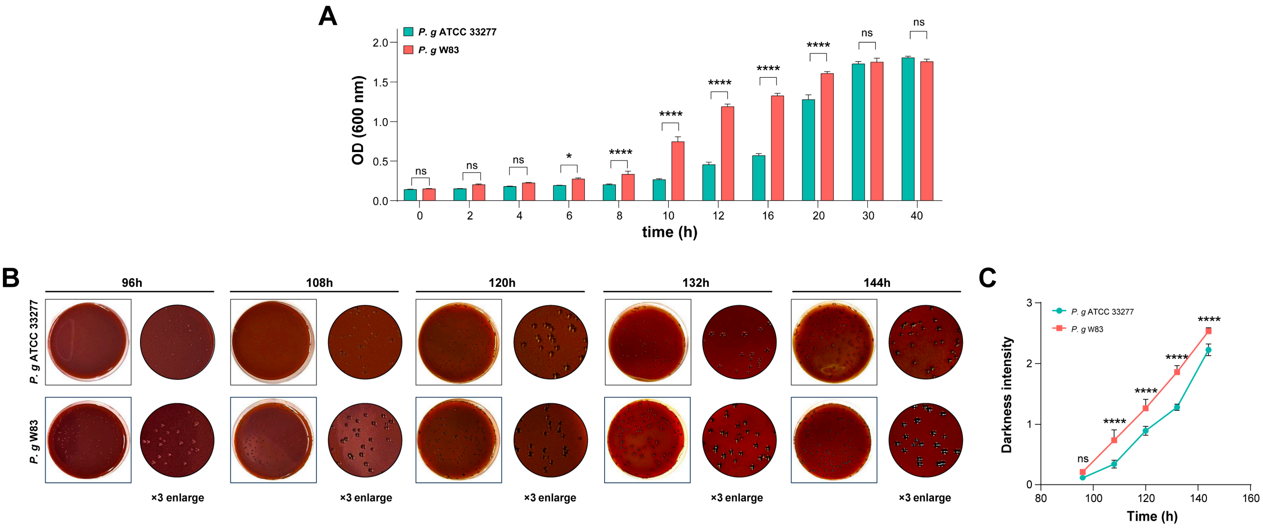

Fig. S3 (**A**) Comparison of growth between *P. gingivalis* ATCC 33277 and W83 at 0, 2, 4, 6, 8, 10, 12, 16, 20, 30, and 40 h. (**B**) Representative images of colony pigmentation on blood agar plates after 96-144 h of anaerobic incubation. (**C**) Quantitative analysis of Fig. S3B. Statistical significance: ns, not significant; **P* < 0.05, ***P* < 0.01, ****P* < 0.001, and *****P* < 0.0001. < 0.0001.

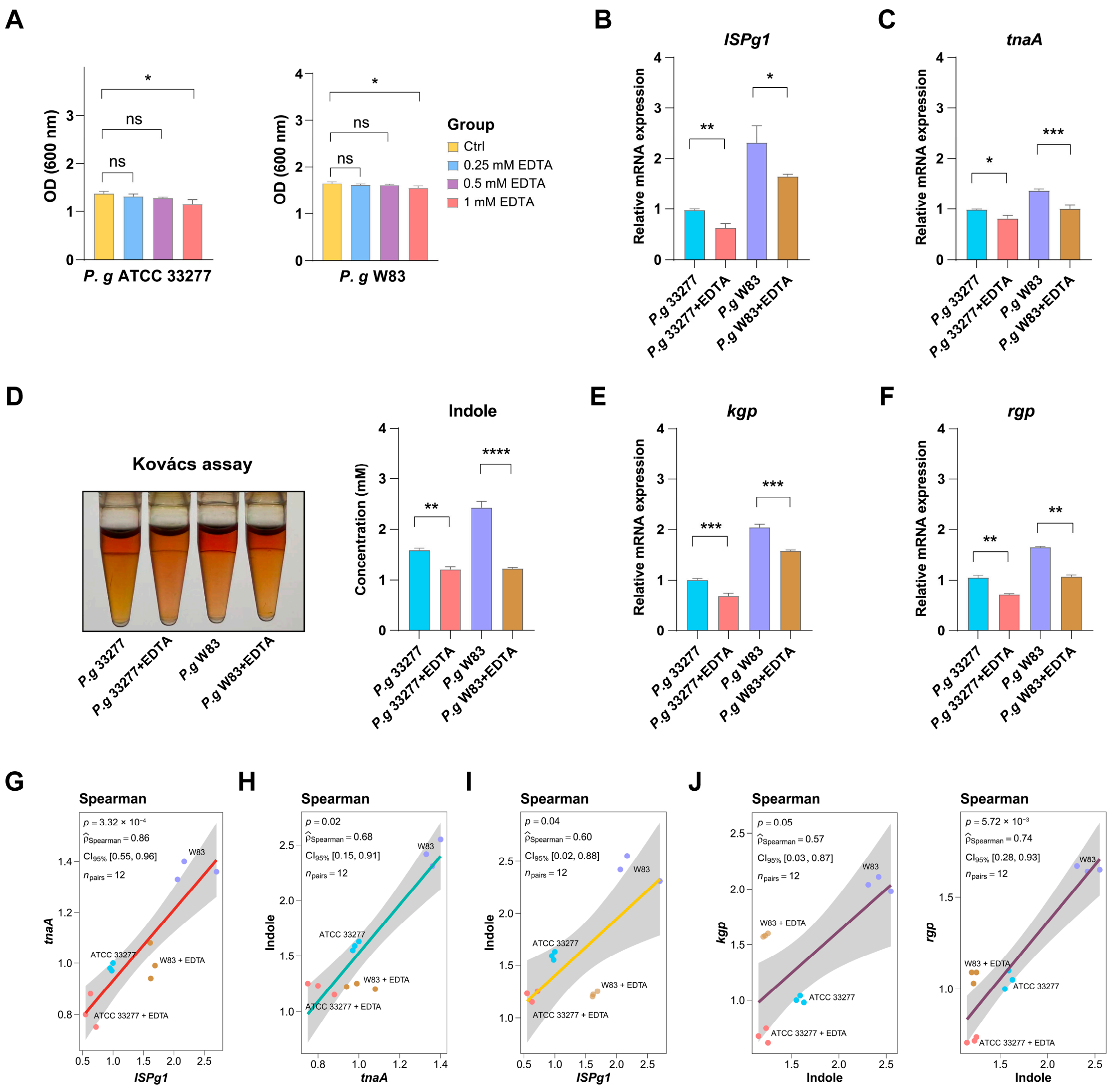

Fig. S4 (**A**) Effect of EDTA concentrations on *P. gingivalis* growth. The concentration of 0.5 mM was cho-sen for the subsequent experiments. (**B,C**) Relative mRNA expression of *ISPg1* and *tnaA*. (**D**) Kovács assay (left) and quantitative detection (right). (**E,F**) Relative mRNA expression of *kgp* and rgp. (**G-J**) Spearman correlation analysis between *ISPg1* and *tnaA*, *tnaA* and indole, *ISPg1* and indole, indole and *kgp*/*rgp*. Shaded areas: 95% confidence intervals. Statistical significance: ns, not significant; **P* < 0.05, ***P* < 0.01, ****P* < 0.001, and *****P* < 0.0001.
